# Digital, DICOM, Diagnostik – Einheit statt Chaos: Datenaustausch in der digitalen Pathologie

**DOI:** 10.1007/s00292-025-01511-0

**Published:** 2025-11-29

**Authors:** Christoph Blattgerste, Maximilian Legnar, Cleo-Aron Weis

**Affiliations:** 1 Computational Pathology Heidelberg, Institut für Pathologie, Uniklinikum Heidelberg, Im Neuenheimer Feld 224, 69120 Heidelberg, Deutschland; 2 Medizinische Fakultät Heidelberg, Universität Heidelberg, Heidelberg, Deutschland

**Keywords:** Computergestützte Pathologie, „Whole slide images“, Datenkommunikation, Computer Vision, Standardisierung, Computational pathology, Whole slide images, Data communication, Computer vision, Standardization

## Abstract

Die Digitalisierung histologischer Schnitte eröffnet neue Potenziale für die KI-gestützte Diagnostik, wird jedoch durch fehlende Standards behindert. Besonders die Interoperabilität zwischen proprietären Formaten, Algorithmen und klinischen Systemen wie PACS oder LIS stellt eine zentrale Herausforderung dar. Das interdisziplinäre DICOM-Format bietet hierfür eine offene, herstellerunabhängige Lösung, die Bilddaten, Metadaten und Analyseergebnisse integriert und interoperablen Austausch ermöglicht. Eine durchgeführte Literaturrecherche zeigt eine wachsende Zahl an Publikationen zur digitalen und computergestützten Pathologie, wobei DICOM zunehmend als Schlüsselformat diskutiert wird. Mit einer quelloffenen, modularen Docker-Pipeline demonstrieren wir die praktische Umsetzung DICOM-konformer Workflows zur Speicherung und Visualisierung von „whole slide images“ und KI-Ergebnissen. Damit wird die Basis für standardisierte, transparente und vertrauenswürdige digitale Pathologie geschaffen.

**Graphic abstract:**

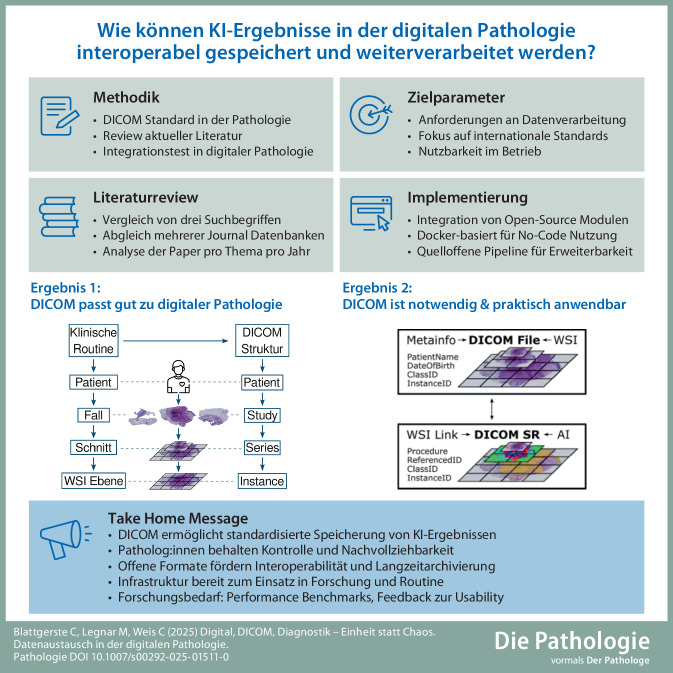

## Abstract (en)

Digitization of histological specimens enables new computer-assisted analysis and artificial intelligence (AI)-supported diagnostics, but is hampered by a lack of standards. Interoperability between proprietary formats and clinical systems such as the Picture Archiving and Communication System (PACS) or Laboratory Information Systems (LIS) poses a particular challenge. The Digital Imaging and COmmunications in Medicine (DICOM) format, adapted from radiology, offers an open, vendor-independent solution that integrates image data, metadata, and analysis results and enables interoperable exchange. A literature review shows a growing number of publications on digital and computer-assisted pathology, with DICOM increasingly being discussed as a key format. With an open-source, modular Docker pipeline, we demonstrate the practical implementation of DICOM-compliant workflows for storing and visualizing whole slide images and AI results. This creates the basis for standardized, transparent, and trustworthy digital pathology.

## Hintergrund

Mit der digitalen Pathologie entstehen neue Anwendungen, die das Fach grundlegend verändern. Die Digitalisierung histologischer Schnitte ermöglicht nicht nur effiziente Archivierung, sondern auch computergestützte Bildanalyse und KI-gestützte Assistenzsysteme. Ein Hindernis für die breite Einführung sind fehlende Standards. Besonders problematisch ist die mangelnde Interoperabilität zwischen proprietären Bildformaten und klinischen Systemen wie PACS („Picture Archiving and Communication System“) und LIS (Labor-Informations-Systeme).

Hier setzt das für die Pathologie adaptierte DICOM-Format („Digital Imaging and COmmunications in Medicine“) an. Als offener Standard ermöglicht es die gemeinsame Speicherung von Bilddaten, Metadaten und Analyseergebnissen wie Segmentierungen oder Klassifikationen. Es unterstützt den Austausch zwischen PACS, LIS und Viewern, erlaubt langfristige Archivierung und integriert Datenschutzfunktionen [1]. Ziel dieses Beitrags ist ein Überblick zu aktuellen Entwicklungen mit Fokus auf DICOM als Austauschformat für „Whole Slide Images“ (WSI) und zugehörige Analysen. Ergänzend wird eine quelloffene Pipeline vorgestellt, die DICOM-konform KI-Ergebnisse speichert und visualisiert.

**Abb. 1:**
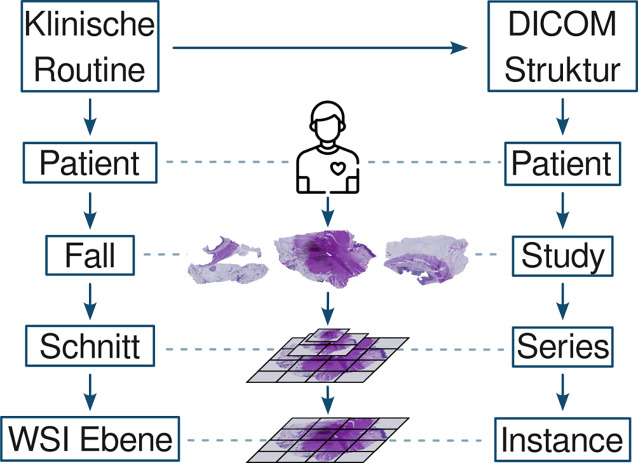
Die Einteilung von Gewebeschnitten in der Pathologie ist dem Aufbau der DICOM-Architektur („digital imaging and communications in medicine“) sehr ähnlich und ermöglicht damit eine problemlose Abbildung der Struktur

**Abb. 2:**
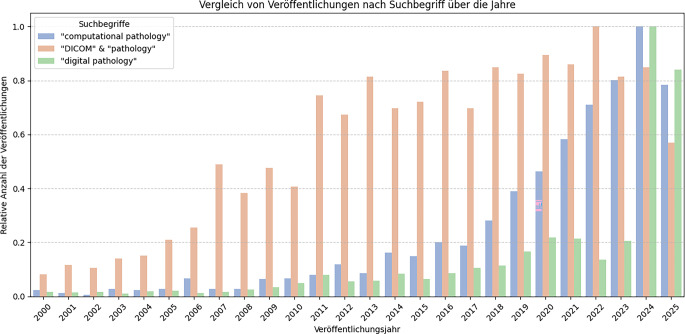
Verglichen werden Veröffentlichungen für die Suchbegriffe „Computational Pathology“, „Digital Pathology“ und „DICOM & Pathology“ („Digital Imaging and COmmunications in Medicine“) nach ihrem Erscheinungsjahr. Einbezogen wurden jeweils 1000 Suchergebnisse von Semantic Scholar, ArXiv und PubMed für jeden Suchbegriff. Ergebnisse für das laufende Jahr 2025 wurden bis August einbezogen

Verglichen mit der pathologischen Nomenklatur zeigt Abb. 1 die passende DICOM-Struktur: „patient, study, series, instanz“ (Abb. 2). Eine DICOM-Instanz fungiert als Container für Bilddaten, Patienteninformationen und Befunde wie detailliert von D. Clunie beschrieben [1]. Besonders wichtig sind Erweiterungen der Dateiarchitektur sowie der DICOMweb-Spezifikation, die REST-Schnittstellen („REpresentational State Transfer“) für Lesen, Bearbeiten und Schreiben definieren. Digitale Pathologie erfordert dabei besondere Lösungen für Speicherung, Visualisierung und Annotation sehr großer WSI-Dateien. Erst durch die Arbeit spezialisierter Fachgruppen konnten radiologische PACS-Systeme auf Pathologie erweitert werden.

**Abb. 3:**
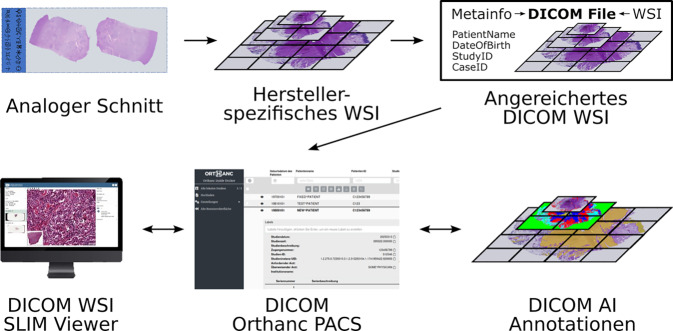
Pipeline der digitalen Pathologie implementiert den WSIDicomizer von IMI Bigpicture, das Picture Archive & Communication System von Orthanc sowie den Slim Viewer für Whole Slide Images

## Methode

Zur systematischen Erfassung des Standes der Technik wurde eine automatisierte Literaturrecherche durchgeführt. Ein Python-Skript fragte für die Begriffe „Digital Pathology“, „Computational Pathology“ und „DICOM & Pathology“ jeweils die 1000 passendsten Manuskripte aus PubMed, ArXiv und Semantic Scholar über die internen Suchfunktionen ab. So konnten technische und medizinische Publikationen und ihre Metadaten zusammengeführt werden. Anhand hinreichend großer Stichproben der Datenbanken erfolgte die Analyse anhand relativer Häufigkeiten pro Jahr (Abb. 2).

## Stand der Technik

Bevor die Entwicklung quantitativ analysiert wird, ist eine qualitative Bestandsaufnahme angebracht.

Open-source-Tools wie QuPath, OMERO oder SlideViewer [4] sowie kommerzielle Lösungen unterstützen bereits DICOM für die Pathologie [6]. Darüber hinaus implementiert die DICOMweb-Schnittstelle moderne RESTful API für Abfragen, Speicherung und Zugriff auf Objekte [1]. Dennoch exportieren die meisten Scanner proprietäre Formate. Auch wenn erste Geräte ab Werk DICOM nutzen, ist häufig eine Konvertierung nötig, um Interoperabilität zu erreichen. Die fehlende Standardisierung erschwert plattformübergreifenden Austausch, Archivierung und wissenschaftliche Vergleichbarkeit.

## Ergebnisse

Die quantitative Literatursuche bestätigt die anfangs erläuterten Bedarfe digitaler Pathologie verbunden mit den Anforderungen von computergestützter Pathologie.

Zu beiden Themenbereichen sind über alle drei Suchdatenbanken über die letzten Jahre mehr und mehr Veröffentlichungen erschienen. Dabei ist zu sehen, dass die digitale Pathologie bereits vor der Computeranalyse von Interesse war. Dies kann an der Notwendigkeit der Digitalisierung liegen, bevor digitale Bilddaten analysiert werden können. Oder auch, eine computergestützte Arbeitsweise in der Pathologie war erst mit digitalen WSI möglich und erforschbar. Keinesfalls ist ein Abfall der Veröffentlichungen zu digitaler Pathologie zu beobachten, womit nach wie vor innovative Forschung und Entwicklung in diesem Themenfeld betrieben wird. Die Kombination der Suchbegriffe von DICOM und Pathologie ergibt eine seit vielen Jahren hohe Zahl von Publikationen mit weiterhin steigender Tendenz. Auch beide referenzierten Artikel von D. Clunie [1] und M. Romanchikova et al. [2] beschreiben detailliert die gute Passform von DICOM auf pathologische Abläufe. Die zunehmende Zahl von Veröffentlichungen im Bereich computergestützter Methoden hat keinen Rückgang der Publikationen zu DICOM und digitaler Infrastruktur zur Folge. Somit ist nicht zu beobachten, dass die für AI basierte Analytik wichtige Datenübertragung bereits vollends ausgereift oder optimiert ist, wie die Studie von M. Romanchikova et al. [2] ebenfalls aufzeigt. Insgesamt ist Deutschland basierend auf den Affiliations der Erstautoren auf Platz drei hinter den Vereinigen Staaten von Amerika (USA) und China. Auch wenn der DICOM-Standard in den USA entwickelt wurde, wurden diese Entwicklungen in Deutschland aufgenommen und erfolgreich weitererforscht.

Die Auswertung verschiedener Quellen [1, 2] und Publikationsdatenbanken zeigt, dass das DICOM-Format zentrale Anforderungen an eine standardisierte Datenhaltung in der digitalen Pathologie erfüllt. DICOM vereint heterogene Datenquellen, ermöglicht interoperablen Austausch und unterstützt multimodale Bildanalyse, wobei diese Vorteile besonders bei der automatisierten, computer- und KI-gestützten Datenanalyse von Bedeutung sind [6].

Trotz dieser Stärken bestehen in der praktischen Anwendung noch Hürden. Die meisten Scanner exportieren WSI weiterhin in proprietären Formaten, und viele gängige Viewer unterstützen DICOM-basierte WSI nicht vollständig. Auch Performanceprobleme bei großen Bilddaten sowie eine fehlende Unterstützung strukturierter Annotationen im „DICOM structured reporting“ (SR) behindern die unmittelbare klinische Nutzung [2].

## Implementierung

Zur Demonstration wurde eine modulare Open-source-Pipeline, wie in Abb. 3 gezeigt, entwickelt: https://github.com/cpheidelberg/Patho-DICOM-Pipeline. Sie basiert auf Docker, umfasst den Orthanc PACS-Server [3], den browserbasierten Slim Viewer [4] sowie den WSIDicomizer zur Konvertierung proprietärer Dateien. KI-Ergebnisse werden als strukturierte DICOM-SR-Objekte eingebettet. Optional kann ein BabelFish-basiertes Tool [5] für Slide-Identifikation und Fallverknüpfung integriert werden.

Die Implementierung zeigt: DICOM-basierte Systeme können Forschung, Lehre und klinische Anforderungen verbinden. Gleichzeitig wird deutlich, dass stärkere Unterstützung durch Hersteller und Standardisierungsgremien nötig ist, um diese Ansätze in die Routine zu überführen. Erste Anbieter wie Sectra setzen DICOM bereits als universellen Standard ein (Rålund, Sectra).

## Fazit

- Die vorliegende Arbeit zeigt, dass der offene DICOM-Standard („digital imaging and communications in medicine“) eine zuverlässige Grundlage für die Integration von KI-Ergebnissen in der digitalen Pathologie bietet.
- Durch die strukturierte Einbettung von Analyseergebnissen und Metadaten lassen sich Bilddaten, Analysen und Patienteninformationen nahtlos verknüpfen. Die entwickelte, quelloffene Docker-Pipeline demonstriert die technische Machbarkeit und ermöglicht die Nachnutzung in Forschung und Klinik (https://github.com/cpheidelberg/Patho-DICOM-Pipeline).
- Standards wie DICOM fördern Interoperabilität, Nachvollziehbarkeit und Vertrauen in KI-gestützte Prozesse.
- Für eine flächendeckende Umsetzung bedarf es jedoch weiterer Entwicklungsarbeit an der Einbindung in Archivierungs- und Viewer-Systemen, Annotationstools und Klinikalltag.
- Besonders eine enge Zusammenarbeit zwischen Forschung, Industrie und klinischer Praxis ist entscheidend, um nachhaltig digitale Infrastrukturen in der Pathologie zu etablieren.
